# Supplementary figures and images for: Epigenetic instability may alter cell state transitions and anticancer drug resistance
Source: PLoS Comput Biol. 2021 Aug 23;17(8):e1009307. doi: 10.1371/journal.pcbi.1009307 (PMC8412323; doi:10.1371/journal.pcbi.1009307)

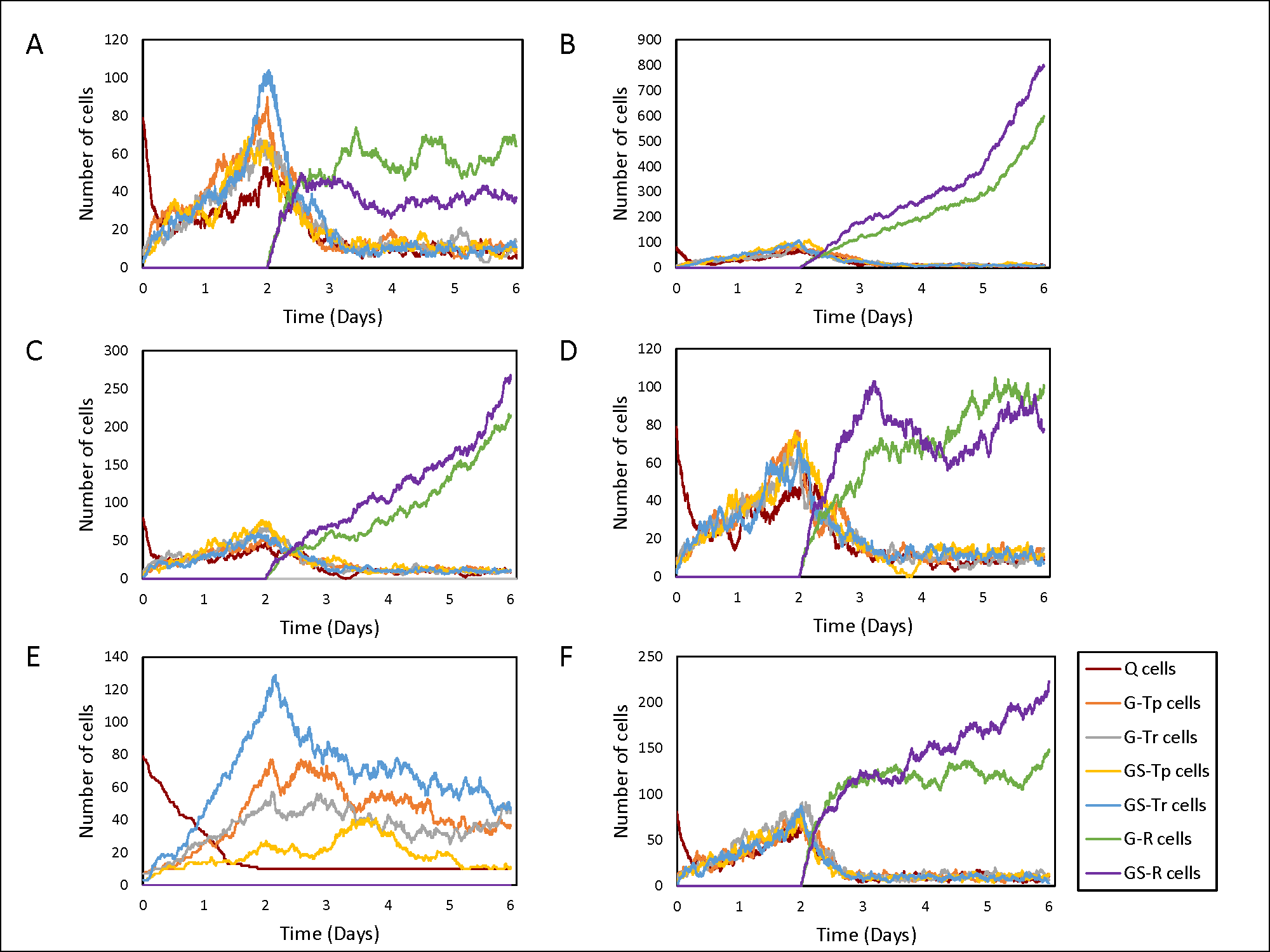

Supplement: S1 Fig — (A) Simulation for low birth rate of resistant cells (1.5*n). (B) Simulation for high birth rate of resistant cells (2.5*n). (C) Simulation for low death rate of resistant cells (2.2*n* e-(t- 2)/10). (D) Simulation for high death rate of resistant cells (2.2*n* e-(t- 2)/100). (E) Simulation without any transition. (F) Simulation with high transition rate (2*n). (TIFF) [file pcbi.1009307.s001.tiff]

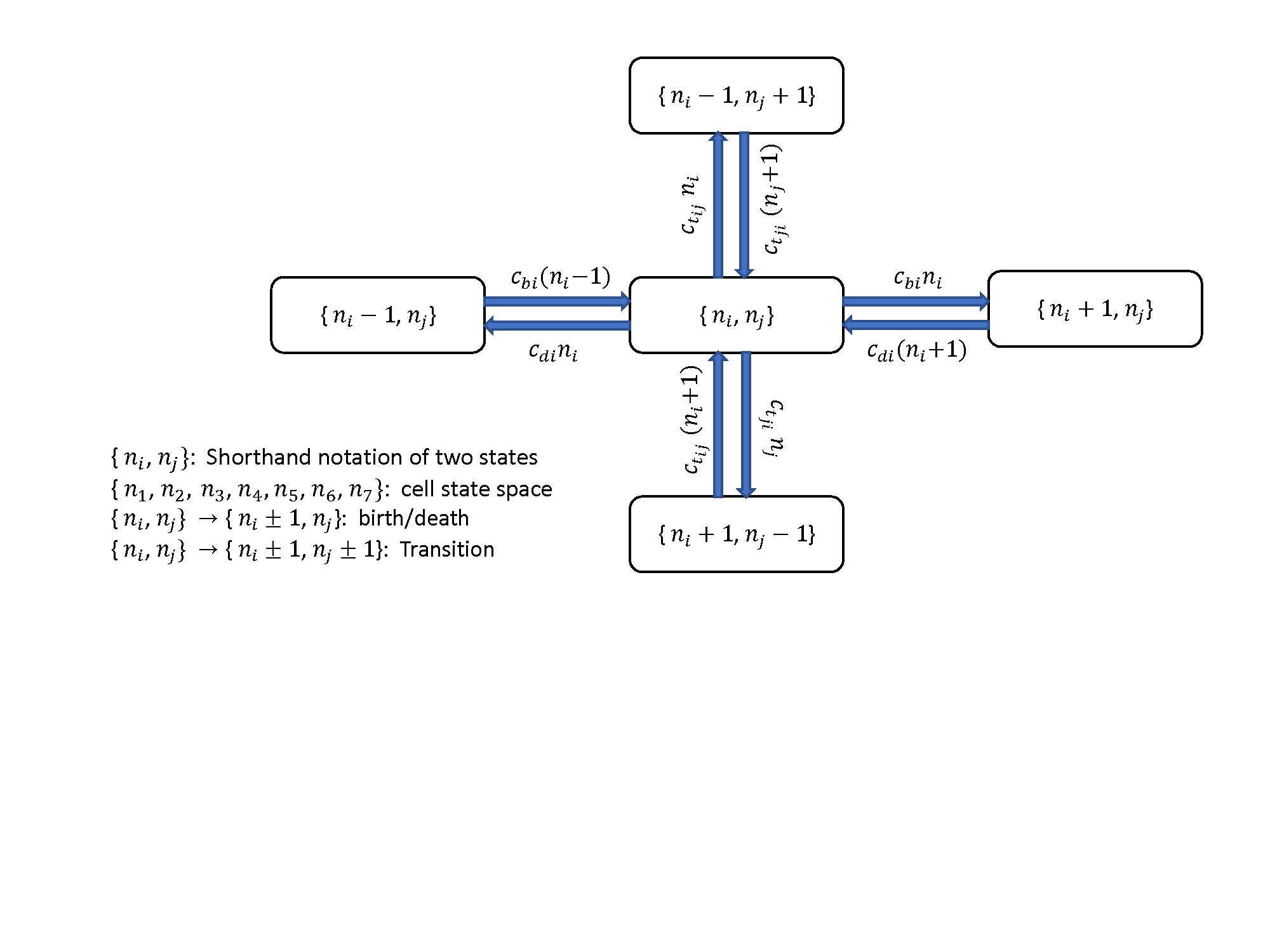

Supplement: S2 Fig — Illustration of the flow of cell state probability within the cell state space through the process of birth, death, and transition. ni Indicates the number of cells in the ith state. cbi, cdi and ctij are birth, death and transition rate coefficient, respectively. (TIF) [file pcbi.1009307.s002.tif]

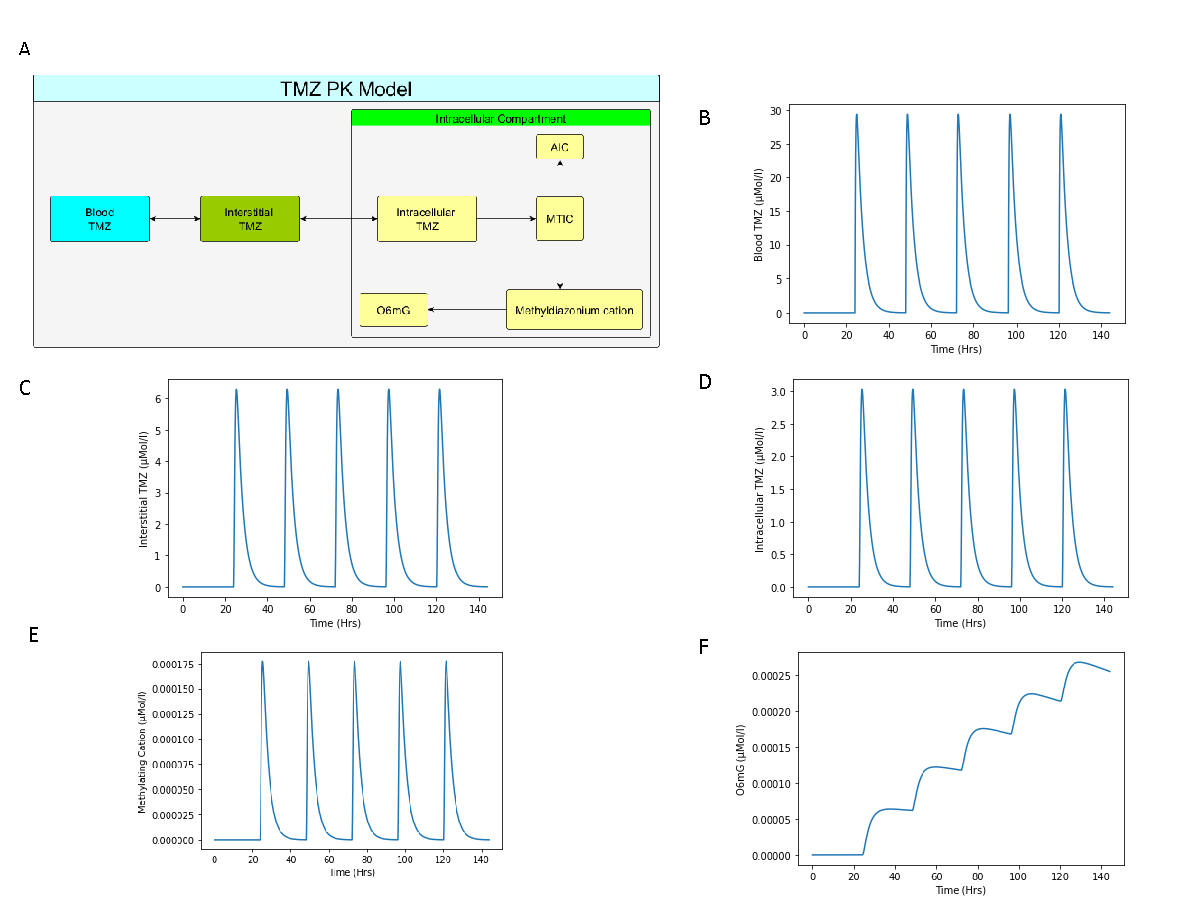

Supplement: S3 Fig — (A) The TMZ PK model based on that developed by Ballesta et al [1] that shows the intracellular metabolic conversion from TMZ to O6mG. An oral 150 mg/m2 TMZ dose every 24 hours for 5 days starting at t = 24 hours produced concentration-time profiles of TMZ in blood (B), interstitial fluid TMZ (C), intracellular TMZ (D), methylating cation (E), and O6mG (F). The O6mG concentrations are greater than in Fig 6 since there is no MGMT repair considered here. (TIFF) [file pcbi.1009307.s003.tiff]
