# Supplementary material for: Epigenetic instability may alter cell state transitions and anticancer drug resistance
Source: PLoS Comput Biol. 2021 Aug 23;17(8):e1009307. doi: 10.1371/journal.pcbi.1009307 (PMC8412323; doi:10.1371/journal.pcbi.1009307)
Supplement: S1 Text — (DOCX) [file pcbi.1009307.s004.docx]

**Supplement**

**1. Effect of birth, death and transition rates in drug resistant cells**

Different cell birth, death and transition rates were examined to assess their effect on population dynamics (Table A in S1 Text). A lower birth rate (1.5*n) led to a smaller resistant cell population in comparison to a higher birth rate (2.5*n) (S1A and S1B Fig). To illustrate the effect of drug-induced time dependence on the death rate of resistant cells, two different death rates (e^-(t- 2)/10^ and e^-(t- 2)/100^) were used. In the first case (e^-(t- 2)/10^), the death rate decreased rapidly leading to an exponential rise of resistant cell population, whereas in the second case (e^-(t- 2)/100^), the death rate decreased slowly, and thus resistant cells died at a faster rate (S1C and S1D Fig).

**S1 Fig: Effects of changing birth/death/transition rates on cell population dynamics.**

(A) Simulation for low birth rate of resistant cells (1.5*n). (B) Simulation for high birth rate of resistant cells (2.5*n). (C) Simulation for low death rate of resistant cells (2.2*n* e^-(t- 2)/10^). (D) Simulation for high death rate of resistant cells (2.2*n* e^-(t- 2)/100^). (E) Simulation without any transition. (F) Simulation with high transition rate (2*n).

We next considered cell states under two conditions; either having no transitions or having high transition rates (2*n). In the absence of transitions, there is no resistant cell population as resistant cells arise from sensitive cells. Additionally, the cell state trajectories remain independent of each other resulting in large disparity in the number of cells occupying different cell states (S1E Fig). In the case of high transition rates, resistant cells dominated the cell population. However, allowing transitions between cells states changed the behavior of the system by allowing the cells to move from more populated states to lesser-populated states (S1F Fig). Hence, most of the cell state trajectories remained close to each other.

| **Condition** | **Time**  **(t)** | **Q Cells** | | | **G-Tp, G-Tr**  **GS-Tp, GS-Tr** | | | **G-R, GS-R** | | | **Figure Number** |
| --- | --- | --- | --- | --- | --- | --- | --- | --- | --- | --- | --- |
|  |  | **BR** | **DR** | **TR** | **BR** | **DR** | **TR** | **BR** | **DR** | **TR** |  |
| Low BR of G-R, GS-R | t < 2 | 0 | n | n | 2*n | n | n | NA | NA | NA | S1A Fig |
|  | t > 2 | 0 | 2.2*n | n | 2*n | 2.2*n | n | 1.5*n | 2.2*n* e^-(t- 2)/20^ | n |  |
| High BR of G-R, GS-R | t < 2 | 0 | n | n | 2*n | n | n | NA | NA | NA | S1B Fig |
|  | t > 2 | 0 | 2.2*n | n | 2*n | 2.2*n | n | 2.5*n | 2.2*n* e^-(t- 2)/20^ | n |  |
| Low DR of G-R, GS-R | t < 2 | 0 | n | n | 2*n | n | n | NA | NA | NA | S1C Fig |
|  | t > 2 | 0 | 2.2*n | n | 2*n | 2.2*n | n | 2*n | 2.2*n* e^-(t- 2)/10^ | n |  |
| High DR of G-R, GS-R | t < 2 | 0 | n | n | 2*n | n | n | NA | NA | NA | S1D Fig |
|  | t > 2 | 0 | 2.2*n | n | 2*n | 2.2*n | n | 2*n | 2.2*n*e^-(t- 2)/100^ | n |  |
| No Transition rate | t < 2 | 0 | n | 0 | 2*n | n | 0 | NA | NA | NA | S1E Fig |
|  | t > 2 | 0 | 2.2*n | 0 | 2*n | 2.2*n | 0 | 2*n | 2.2*n* e^-(t- 2)/20^ | 0 |  |
| High Transi-  -tion rate | t < 2 | 0 | n | 2*n | 2*n | n | 2*n | NA | NA | NA | S1F Fig |
|  | t > 2 | 0 | 2.2*n | 2*n | 2*n | 2.2*n | 2*n | 2*n | 2.2*n* e^-(t- 2)/20^ | 2*n |  |

**Table A:** Summary of model simulation conditions with alternate birth, death, and transition rates. n indicates the number of cells in a state; t is the time elapsed in the system. BR: Birth rate, DR: Death rate and TR: transition rate.

**2. Mathematical description of the model**

As stated before, in our model, there are seven cell states, which are denoted as a row vector${\{n}_{1},n_{2},..,n_{7}\}$ ($n_{i}$ represent the number of cells in the *i*^th^ state). We assume continuous time Markovian dynamics for cell proliferation, which means that the future evolution of the system can be predicted from the current state alone (memoryless). The process of birth, death, and transition between any cell states $i$ and $j$ can be written as

Birth: $n_{i} \underset{\to}{c_{bi}} n_{i}+1$ Death: $n_{i} \underset{\to}{c_{di}} n_{i}-1$

Transitions: $n_{i} \underset{\to}{c_{t_{ij}}} n_{i}-1 n_{j} \underset{\to}{c_{t_{ij}}} n_{j}+1$

where $c_{bi}$ and $c_{di}$ is the birth and death rate coefficient in $i^{th}$ cell state, respectively. $c_{t_{ij}}$ is the transition rate coefficient regarding the transition from $i^{th}\to j^{th}$ cell state. All of the above processes can be written in terms of transitional probabilities (S2 Fig) as

**S2 Fig: Cell state probability flowchart.**

Illustration of the flow of cell state probability within the cell state space through the process of birth, death, and transition. $n_{i}$ Indicates the number of cells in the $i^{th}$ state. $c_{bi}$, $c_{di}$ and $c_{t_{ij}}$ are birth, death and transition rate coefficient, respectively.

Birth: $P\left( n_{1}+1,n_{2},..,n_{7}; t+\Delta t \mid n_{1},n_{2},..,n_{7}; t \right)=c_{b1}n_{1} \Delta t$

$P\left( n_{1},n_{2}+1,..,n_{7}; t+\Delta t \mid n_{1},n_{2},..,n_{7}; t \right)=c_{b2}n_{2} \Delta t$

.

.

.

$$P\left( n_{1},n_{2},..,n_{7}+1; t+\Delta t \mid n_{1},n_{2},..,n_{7}; t \right)=c_{b7}n_{7} \Delta t$$

Death: $P\left( n_{1}-1,n_{2},..,n_{7}; t+\Delta t \mid n_{1},n_{2},..,n_{7}; t \right)=c_{d1}n_{1} \Delta t$

$P\left( n_{1},n_{2}-1,..,n_{7}; t+\Delta t \mid n_{1},n_{2},..,n_{7}; t \right)=c_{d2}n_{2} \Delta t$

.

.

.

$$P\left( n_{1},n_{2},..,n_{7}-1; t+\Delta t \mid n_{1},n_{2},..,n_{7}; t \right)=c_{d7}n_{7} \Delta t$$

Transitions: $P\left( n_{1}-1,n_{2}+1,..,n_{7}; t+\Delta t \mid n_{1},n_{2},..,n_{7}; t \right)=c_{t_{12}}n_{1} \Delta t$

$P\left( n_{1}+1,n_{2}-1,..,n_{7}; t+\Delta t \mid n_{1},n_{2},..,n_{7}; t \right)=c_{t_{21}}n_{2} \Delta t$

.

.

.

$$P\left( n_{1},..,n_{6}-1,n_{7}+1; t+\Delta t \mid n_{1},n_{2},..,n_{7}; t \right)=c_{t_{67}}n_{6} \Delta t$$

$P\left( n_{1},\ldots, n_{6}+1,n_{7}-1; t+\Delta t \mid n_{1},n_{2},..,n_{7}; t \right)=c_{t_{76}}n_{7} \Delta t$

In stochastic systems, time evolution is given by a master equation, which is derived by supposing that the system is in state $\{n_{1},\ldots, n_{6},n_{7}\}$ at time t, and based on the transitions defined above, we can write the probability distribution of the state $\{n_{1},\ldots, n_{6},n_{7}\}$ at time $t+\Delta t$ as

$P\left( n_{1},\ldots, n_{6},n_{7}; t+\Delta t \right)=c_{b1}\left( n_{1}-1 \right)\Delta t P\left( n_{1}-1,\ldots, n_{6},n_{7}; t \right)+ c_{b2}\left( n_{2}-1 \right)\Delta t P\left( n_{1},n_{2}-1,\ldots,n_{7}; t \right)+\ldots+ c_{d1}\left( n_{1}+1 \right)\Delta t P\left( n_{1}+1,\ldots, n_{6},n_{7}; t \right)+ c_{d2}\left( n_{2}+1 \right)\Delta t P\left( n_{1},n_{2}+1,\ldots,n_{7}; t \right)+\ldots+ c_{t_{12}}\left( n_{1}+1 \right)\Delta t P\left( n_{1}+1,n_{2}-1,..,n_{7}; t \right)+ c_{t_{21}}\left( n_{2}+1 \right)\Delta t P\left( n_{1}-1,n_{2}+1,..,n_{7}; t \right)+ c_{t_{13}}\left( n_{1}+1 \right)\Delta t P\left( n_{1}+1,n_{2}, n_{3}-1,..,n_{7}; t \right)+ c_{t_{31}}\left( n_{3}+1 \right)\Delta t P\left( n_{1}-1,n_{2}, n_{3}+1,..,n_{7}; t \right)+\ldots+\left( 1-\alpha\right)\Delta t P\left( n_{1},\ldots, n_{6},n_{7}; t \right)$

where $\alpha$ is the sum of all transition probabilities from other states to the state $\{n_{1},\ldots, n_{6},n_{7}\}$. The above equation can be written in a succinct form as

$$P\left( n_{1},\ldots, n_{6},n_{7}; t+\Delta t \right)=\sum_{i} c_{bi}\left( n_{i}-1 \right)\Delta t P\left( n_{i}-1; t \right)+\sum_{i} c_{di}\left( n_{i}+1 \right)\Delta t P\left( n_{i}+1; t \right)+\sum_{i, j} c_{t_{ij}}\left( n_{i}+1 \right)\Delta t P\left( n_{i}+1,n_{j}-1 ; t \right)+\left( 1-\alpha\right)\Delta tP\left( n_{1},\ldots, n_{6},n_{7}; t \right)$$

Applying a few algebraic manipulations yields the master equation as

$\frac{dP(n_{i})}{dt}=\sum_{i} c_{bi}\left( n_{i}-1 \right)P\left( n_{i}-1; t \right)+\sum_{i} c_{di}\left( n_{i}+1 \right)P\left( n_{i}+1; t \right)+\sum_{i, j} c_{t_{ij}}\left( n_{i}+1 \right)P\left( n_{i}+1,n_{j}-1 ; t \right)-\alpha P\left( n_{1},\ldots, n_{6},n_{7}; t \right)$ Eq. (S1)

The first term corresponds to the birth, the second term to death, the third term represents outward and inward transitions from and to a particular state, and the last term is the probability of none of the above events occurring. Both birth and death rates are proportional to the number of cells in the corresponding state. The transition rate is proportional to the number of cells in the initial state ($n_{i}$). The coefficients are set according to the birth rates, death rates, and transition rates between the states. Once the initial values of ($n_{i}$) and coefficients ($c_{bi},c_{di}, c_{t_{ij}}$) are known, the stochastic time evolution of the system can be evaluated using the master equation.

**3. Simulations**

To simulate the system, the Gillespie algorithm was used, where individual trajectories are simulated instead of the whole probability distribution. The algorithm assumes the processes to be Markovian and a time step ($\tau$) is used based on the probability of the event occurrence (Poisson distribution). To find $\tau$, we define the propensity function as

$a_{0}=\sum_{i} a_{i}$ Eq. (S2)

where the probability that any given event will occur per unit time is $a_{i}$. The time before the next reaction occurs is a random variable with distribution

$p\left( \tau\right)=a_{0}e^{-a_{0}\tau}$ Eq. (S3)

We can generate an exponentially distributed τ (time to next reaction) by

$\tau=\frac{1}{a_{0}}ln\left( \frac{1}{r_{1}} \right)$ Eq. (S4)

where,$r_{1}$ is a uniformly distributed random number in (0, 1). We used another random number to select which of the reactions (based on their weight) is going to take place. If event 1 is selected: $n_{1} \underset{\to}{} n_{1}+1$. If event 2 is selected: $n_{1} \underset{\to}{} n_{1}-1$ and so on. We update the number of cells in each cell state and repeat the process until the desired time or maximum cell population is reached. The code is available on GitHub (<https://github.com/anshulsa/Cell_state_transition.git>).

**4. Hybrid Stochastic-Deterministic TMZ-MGMT Model**

The pharmacokinetic (PK) model of TMZ used to simulate O6mG adduct concentrations was based on prior work from our lab that put forth the concept of cell-type specific PK and pharmacodynamic (PD) models [1]. The specific PK model is illustrated in S3 Fig and shows the intracellular metabolic conversion of TMZ to the methylating cation and finally to the O6mG DNA adducts. It was assumed that O6mG represented 5% of the total TMZ-induced DNA adducts (N3-methyladenine and N7-methylguanine adducts are also generated by TMZ) [2]. The typical multiple-dose TMZ regimen used in GBM patients produces blood TMZ concentration-time profiles consistent with prior observations [3]. The MGMT protein production is modeled using two level transcription and translation model with a positive feedback loop (Fig 5) [4]. The O6mG adduct produced due to TMZ is consumed by the MGMT protein. The model consisted of the following set of ODE equations

$\frac{d r_{m}}{dt}= k_{TC}\left( 1+1000*e \right)\left( \frac{{p_{m}}^{n}}{{p_{m_{0}}}^{n} + {p_{m}}^{n}} \right)- \lambda_{r}*r_{m}$ Eq. (S5)

$\frac{d p_{m}}{dt}= k_{TL}*r_{m}- k_{MG}*O6mG*p_{m}- \lambda_{p}*p_{m}$ Eq. (S6) $\frac{d O6mG}{dt}= k_{O6Add}MeC- \lambda_{O6Add}O6mG- k_{MG}*O6mG*p_{m}$ Eq. (S7)

where

$r_{m}$ , $p_{m}$, O6mG and $MeC$ = MGMT mRNA, MGMT protein, O6-methylguanine, and Methylating cation. $p_{m_{0}}$ is the activation coefficient which depend on the promoter strength. When $p_{m_{0}}$ = $p_{m}$ , Hill function reaches its half value. The rate constants are given in the Table B in S1 Text.

**S3 Fig: TMZ PK Model.** (A) The TMZ PK model based on that developed by Ballesta et al [1] that shows the intracellular metabolic conversion from TMZ to O6mG. An oral 150 mg/m^2^ TMZ dose every 24 hours for 5 days starting at t = 24 hours produced concentration-time profiles of TMZ in blood (B), interstitial fluid TMZ (C), intracellular TMZ (D), methylating cation (E), and O6mG (F). The O6mG concentrations are greater than in Fig 6 since there is no MGMT repair considered here.

| **Rate constant** | **Symbol** | **Value** | **Units** |
| --- | --- | --- | --- |
| Basal MGMT transcription rate constant | $k_{TC}$ | $1.87*{10}^{-7}$ | $\frac{\mu Mol}{L.hr}$ |
| MGMT translation rate constant | $k_{TL}$ | 120.08 | ${hr}^{-1}$ |
| MGMT mRNA degradation rate constant | $\lambda_{r}$ | 0.054 | ${hr}^{-1}$ |
| MGMT protein degradation rate constant | $\lambda_{p}$ | 0.02 | ${hr}^{-1}$ |
| O6mG-MGMT complex degradation rate constant | $k_{MG}$ | $23.4*{10}^{2}$ | $\frac{L}{\mu Mol.hr}$ |
| O6mG production rate | $k_{O6Add}$ | 0.09 | ${hr}^{-1}$ |
| O6mG degradation rate | $\lambda_{O6Add}$ | 0.0041 | ${hr}^{-1}$ |
| Hill Coefficient | n | 2 | No units |
| Activation Coefficient | $p_{m_{0}}$ | 0.0031 | $\frac{\mu Mol}{L}$ |

**Table B.** Parameters for the MGMT model (see Fig 5 and equations S5-S7).

The above set of ODEs are solved to calculate the MGMT mRNA, MGMT protein and O6mG concentrations within the cell. The O6mG concentration determines the toxicity level within the cell and thereby is, linked to the cell death rate (DR) that was defined as;

$DR=a*n*\frac{O6mG}{\alpha}$ Eq. (S8)

where $a$ is a constant, $n$ is the number of cells in that cell state and $\alpha$ is the normalization constant which is set to be $5*{10}^{-9} \mu Mol/l$.

**References**

1. Ballesta A, Zhou Q, Zhang X, Lv H, Gallo JM. Multiscale design of cell-type-specific pharmacokinetic/pharmacodynamic models for personalized medicine: application to temozolomide in brain tumors. CPT Pharmacometrics Syst Pharmacol. 2014 Apr 30;3(4):e112. doi: 10.1038/psp.2014.9

2. Lee SY. Temozolomide resistance in glioblastoma multiforme. Genes Dis. 2016 May 11;3(3):198-210. doi: 10.1016/j.gendis.2016.04.007.

3. Portnow J, Badie B, Chen M, Liu A, Blanchard S, Synold TW. The neuropharmacokinetics of temozolomide in patients with resectable brain tumors: potential implications for the current approach to chemoradiation. Clin Cancer Res. 2009 Nov 15;15(22):7092-8. doi: 10.1158/1078-0432.CCR-09-1349.

4. Chen T, He HL, Church GM. Modeling gene expression with differential equations. Pac Symp Biocomput. 1999:29-40.
