# Supplementary material for: Epigenetic instability may alter cell state transitions and anticancer drug resistance
Source: PLoS Comput Biol. 2021 Aug 23;17(8):e1009307. doi: 10.1371/journal.pcbi.1009307 (PMC8412323; doi:10.1371/journal.pcbi.1009307)
Supplement: Attachment — Submitted filename: Response to Reviewers.docx [file pcbi.1009307.s005.docx]

**Responses to Reviewers:**

Our specific responses to each reviewer comments are indicated by “>>” and blue text.

Reviewer #1:

1. The authors should discuss more of the relevant literature in terms of modeling the effects of epigenetic on cell-fate decisions (Miyamoto et al. PLoS Comp Biol 2015; Jia et al. Phys Biol 2019) and its effect in designing epigenetic-targeting drugs (Gunnarsson et al. J Theor Biol 2020). They should discuss conceptual and technical similarities and differences in their work vis-à-vis previous literature.

>> These are valid points and we have now included a more through literature review (see page 4, lines 70-80).

2. Cell-state transitions are also possible during cell division (Tripathi et al. PLoS Comp Biol 2020). How does incorporating such effects alter the behavior of the models shown here?

>> We have referenced the Tripathi et al model (reference 9). Our model permits cell division embodied in the birth rates for each cell type except quiescent cells, and such processes occur even with epigenetic instability.

3. Have the authors investigated the impact of having intermittent therapy (i.e. drug holidays) which has been included as a part of various clinical trials now? (Michor & Beal, Cell 2015)

>> The hybrid stochastic-mechanistic model (see Figures 5-6) utilizes an intermittent temozolomide regimen (daily X 5 doses) consistent with treatment of patients with brain tumors. As illustrated in Figures 6, epigenetic instability results in improved outcomes in terms of lower MGMT (drug-resistant enzyme) and higher O6mG adducts due to TMZ.

4. What is so specific about this model in terms of gliomas, as the authors claim?

>> The addition of the hybrid stochastic-mechanistic TMZ MGMT ODE model is referenced to glioblastoma multiforme (GBM). By also referencing the stochastic models to GBM it keeps the modeling effort consistent. However, the stochastic models and distinguishing of cell states is by themselves generic and could be applied to other cancers.

5. Is the only major difference between the first model and the multi-step model that there is one more intermediate step that needs to be completed before the lock-in? If true, how do the results of the model change when more than one such intermediaries are included? Also, how do the effects of bidirectional transitions aggregate in terms of such n-state multi-state models? Can bidirectional transitions and multi-state modes sufficient to largely delay tumor resurgence?

>> Yes, the multi-step model used in the investigation contained 3 steps. We illustrate below how the number of steps affects the cell populations with and without epigenetic instability. The changes in the cell populations of resistant cells follow the same pattern with more steps leading to greater reductions in resistant cells. We believe future studies may more precisely link such steps to defined mechanisms that will increase options to design drug therapy.

Reviewer #2: Modeling without experimental validation is just modeling.

>> We have not yet undertaken experimental validation of the epigenetic instability strategy. However, with the addition of the hybrid stochastic-mechanistic model experimental confirmation is tangible since specific histone PTMs have been identified that are relevant to MGMT expression.

Reviewer #3: The paper needs to be re-written for certain sections as it is not very clear to the reader at the first instance. Also the proposed model needs to be reconsidered as to how much does this statistical model help in understanding drug resistance with respect to other aspects apart from epigenetic instability. Moreover, citing other published articles on the same lines in the manuscript will be appreciated. The idea is good but the authors need to answer certain questions: 1) Is the concept novel or are their other papers that have been published on the same lines been published earlier? 2) How will epigenetic instability in terms of dysregulation alter the drug resistance mechanism? 3) Have the author thought of doing certain experiments to justify their results in terms of any in vitro drug resistance model and epigenetic instability.

>> We have addressed the identified deficiencies by rewriting sections to clarify and better acknowledge previous work by other investigators (see the Introduction and and Discussion). In response to the above points: 1) We are confident epigenetic instability is a new treatment paradigm although as acknowledged epigenetic modifications are known to impact drug resistance. 2) Possibly the more convincing demonstration that epigenetic instability can alter drug resistance is through the new hybrid cell state – TMZ MGMT ODE model results (see Figures 5-6). 3) We have considered how to experimentally confirm epigenetic instability as a viable treatment. We have had a long collaboration with Dr. Jann Sarkaia, Director of the Translational Neuro-Oncology Center at the Mayo Clinic. His group is interested in this project, and we have referenced some of their related papers as it pertains to TMZ, MGMT and epigenetic variables. Finally, we have discussed how epigenetic modifier therapy could be implemented in the Discussion.

Reviewer #4: Authors focus on the most important problem that affects anticancer therapy which is resistance. In this manuscript, the authors offer a new approach focused on causing epigenetic instability to cancer cells. Authors developed a mathematical model of glioblastoma cells that can transition from one state to another based on cell stresses, such as drug therapy and epigenetic plasticity. According to this model, when epigenetically instability was allowed, the number of resistant cells decreased.

Introduction should be improved:
It is important to give information about the epigenetic modifications that authors will introduce in their model, arguing why they decided to introduce histone post-translational modifications instead of other types of epigenetic modifications (DNA methylation, DNA acetylation, etc.).
Authors should also give information about the stochastic cell state models considering permissive or restrictive states.

Discussion should be improved:
It is also important to discuss up to what point the introduction of histone post-translational modifications will not affect health tissue, or which could be the effect of increasing epigenetic instability in health tissue. It would be interesting that authors present a model where epigenetic instability is introduced and increased in normal cells and to report what happens in these cells.

>> We have attempted to improve both the Introduction and Discussion sections.
In the Introduction we have better acknowledged previous work on cell state transitions. Although our model is focused on PTM of histones rather than DNA we have not excluded the latter conceptually. There has been substantial work on DNA modifying agents in cancer therapy and we felt there is more current work on the discovery and development of epigenetic (histone) modifying agents, which in part has molded our thinking. In fact, epigenetic modifers in GBM is just beginning to be explored. Also, the addition of the hybrid stochastic – deterministic model has provided for a more integrated presentation.

The reviewer raises an important point about the effects of epigenetic instability on normal cells and possible adverse effects, which at this stage we can only acknowledge as a potential limitation. Normal cell toxicity and the proposed beneficial actions of epigenetic instability await experimental validation as acknowledged.
